# The community pharmacists’ opinion regarding pharmacist as immunizers for expanding their role and service in Thailand

**DOI:** 10.1017/S1463423625100376

**Published:** 2025-08-15

**Authors:** Orawan Sae-lim, Anisrin Miranshahid, Sutita Daewha, Shosita Siriwat, Chakriya Ditsarapong

**Affiliations:** Department of Clinical Pharmacy, Faculty of Pharmaceutical Sciences, Prince of Songkla University, Hat Yai, Thailand

**Keywords:** community pharmacists, immunizer, opinion, readiness, vaccination

## Abstract

**Background:**

Many countries have permitted community pharmacists to administer vaccines to increase the immunization rate. The policy of Thailand has recently expanded and permitted pharmacists to play a role in immunization.

**Aim:**

The objective of this study was to survey the opinion and readiness of community pharmacists as immunizers.

**Methods:**

This study was a prospective, mixed-methods questionnaire and semi-structured interview. The study included community pharmacists in Hatyai, Songkhla, Thailand. A Likert scale questionnaire to evaluate readiness, opinions, and barriers to providing vaccines was distributed online. The volunteer pharmacists were interviewed about their opinions, distress, and benefits of vaccination services.

**Findings:**

An online survey was completed by 146 pharmacists, and 12 community pharmacists agreed to be interviewed. More than 65% of respondents agreed that vaccination services in community pharmacies are easily accessible to patients. Approximately 46% of pharmacist respondents were willing to be immunizers, and 45% of respondents showed readiness with the availability of pharmacy space for handling vaccinations, their storage, and disposing of sharp objects. Almost all of the respondents showed readiness with knowledge of adverse events following immunization (AEFI) and management. However, most of the concerns were vaccine administration skills, the conflict with other professionals, and the cost of setup and management. The pharmacists required training in vaccine administration skills before providing the service.

**Conclusions:**

The community pharmacies were willing and ready to provide vaccination services for the National List of Essential Vaccines. Vaccine administration skills were the main barriers to vaccination. The training should be done in faculty classes or workshops.

## Introduction

Immunization is one of the methods for disease prevention and survival improvement. It is confirmed to prevent several transmissible diseases at the individual level and control the outbreak at a broader level (WHO, 2020). The World Health Organization estimates that 2–3 million deaths are prevented globally across all age groups due to vaccinations (McKeirnan and Hanson, 2023). The studies showed pharmacists as immunizers increased the effectiveness of patients’ education, the accessibility of vaccinations, and the vaccination coverage rate (Isenor *et al.*, 2018; Merks *et al.*, 2021). To increase the immunization rate, many countries have permitted community pharmacists to administer vaccines, such as Canada, the United States of America, the United Kingdom, France, Portugal, and Switzerland (Isenor *et al.*, 2018; Turcu-Stiolica *et al.*, 2021; Lindner *et al.*, 2022). In Thailand, pharmacists previously were not authorized by law to administer vaccines. The hospital pharmacist’s roles were supplying vaccine and storage, promoting patients to get vaccines, and monitoring the adverse effects, while the scope of the community pharmacist’s role was vaccine education and engagement.

The problem of the unachieved coverage rate for influenza vaccination in Thailand is due to the delayed vaccine delivery to health service units and human resources (Tipayamongkholgul *et al.*, 2016). The acceptance rate for vaccination increased when patients were recommended by the health care professionals (Kitro *et al.*, 2021). After the emerging COVID-19 pandemic, widespread and rapid immunization in the community has been shown to herd immunity and control infectious disease (WHO, 2020). The role of community pharmacists has expanded to include immunizers in several countries for rapid COVID-19 vaccine coverage (Jarab *et al.*, 2022; Alotaibi *et al.*, 2023). There were enhancements to primary healthcare in urban areas to rapidly distribute and coverage of the COVID-19 vaccine in Thailand. Subsequently, community pharmacists were involved in preparing the vaccine and monitoring the adverse events following immunization (AEFI). In December 2021, the Pharmacy Council of Thailand defined the policy to expand and permit pharmacists to play a role in immunization. Pharmacists, as immunizers, must certify the vaccination system and practice training. Providing and administering vaccinations of community pharmacists is not only for the COVID-19 vaccine but also for vaccines in the National List of Essential Vaccines (The Pharmacy Council of Thailand, 2021). However, there are no initiated vaccination services in community pharmacies currently. Vaccination services in community pharmacies involve injection techniques, handling vaccines, storage, cold chain systems, recording, and cost. Starting a service requires preparation about space, knowledge, and practice skills. The community pharmacist’s readiness to provide vaccination services has not been investigated. Understanding pharmacists’ readiness and concern regarding services would inform the preparation of vaccination services. The objective of this study was to survey the opinion and readiness of community pharmacists as immunizers.

## Methods

We conducted a prospective, mixed-methods online questionnaire and semi-structured interview. The study included community pharmacists in a community pharmacy setting in Hatyai, Songkhla, Thailand. This study was conducted in accordance with the Declaration of Helsinki. Community pharmacists were obtained before included in the study. The study was approved by the Ethics Committee. Faculty of Pharmaceutical Sciences, Prince of Songkla University (No of document: 68108/2562).

In the online questionnaire part, the questionnaires were developed by the authors and validated through testing by experts in the fields of questionnaire development and community pharmacy. The questionnaire consisted of 4 sections: the first section was demographic data (gender, age, education level, type of pharmacy program, years of experience, employee status); the second section assessed the readiness of pharmacists for immunization and vaccination services in community pharmacies; the third section surveyed pharmacists’ opinions; and the fourth section was about barriers to vaccination services in community pharmacies. For the second and third sections, each item was on a 5-point Likert scale that ranged from strongly disagree to strongly agree with statement. Reliability testing was evaluated by 20 pharmacists, with Cronbach’s alpha values of 0.81 and 0.76, respectively. The ordinal scale for the first five barriers was assessed in the fourth section.

In the semi-structured interview part, the open questions were developed to focus on the opinion of the pharmacist as an immunizer, the willingness, the distress and benefit of vaccination services in community pharmacies. The questions were piloted with 2 pharmacists and modified for the final version.

### Data collection

Data was collected between January and April 2023. We did sampling by convenience sampling for all community pharmacies that located in Hatyai, Songkhla, Thailand. Two quick-response (QR) codes consisting of questionnaires and interview registration were sent directly to all community pharmacies from the contact list in the Songkhla Provincial Public Health Office. The first QR code involved an introduction and objective of the study, the consent form contained a statement about protecting the confidentiality of respondents, and online questionnaires. The second QR code for inviting the community pharmacist to the semi-structured interview was independent of the questionnaires. The community pharmacist interested in participating could submit the required information, including telephone number, available time, and preferred method for the interview (online or onsite).

For online questionnaires, the pharmacist scanned the first QR code to approach and self-administered the questionnaires. For an interview, the community pharmacist who registered the information in the second QR code, was invited to a face-to-face interview by time and method for the interview (online or onsite) that appropriated the respondent. Each interview was conducted by 3 interviewers, lasting no more than 30 min, and recorded on an audio recorder. The interview ended with data collection when saturation was accessed.

### Statistical analysis

Questionnaires responses were analyzed and reported as numbers and percentages. All interview data was translated to English, double-checked for accuracy in both Thai and English, prior to analysis. Content analysis of the interview data was manually interpreted keyword, coded and reviewed by two independent researchers to examine 3 themes (1. Benefits of the vaccination service, 2. concerns of the vaccination service, and 3. proposing of the vaccination service).

## Results

The questionnaire was sent to 197 community pharmacists in Hatyai, Songkhla, Thailand, and 146 pharmacists completed the survey (Table 1). The overall response rate was 74.1%. The majority of community pharmacists were 30–50 years old (60.4%) and graduated from the Industrial pharmacy program (61.3%). Most of them (49.1%) were pharmacy owners, and 41.5% of respondents had 1–5 years’ experience in pharmacy. Most of the respondents (65.1%) did not know the policy of the Pharmacy Council of Thailand to expand and permit the role of pharmacists in immunization.

**Table 1.** Baseline characteristics (*n* = 146)

	Number (%)
Female	99 (67.9%)
Age	
< 30 years	41 (28.3%)
30–50 years	88 (60.4%)
> 50 years	17 (11.3%)
Education level	
Bachelor’s degree	128 (87.7%)
Master’s degree	18 (12.3%)
Pharmacy program	
Pharmaceutical care	56 (38.7%)
Industrial pharmacy	90 (61.3%)
Experience in pharmacy (year)	
< 1	7 (4.7%)
1–5	61 (41.5%)
6–10	40 (27.4%)
> 10	39 (26.4%)
Working time (hours/week)	
< 20	26 (17.9%)
20–40	19 (13.2%)
41–70	70 (48.1%)
> 70	30 (20.8%)
Employment status	
Pharmacy owner	72 (49.1%)
Full time staff pharmacist	51 (34.9%)
Part time staff pharmacist	23 (16.0%)
Policy to expand and permit the role of pharmacists in immunization	
Known	51 (34.9%)
Unknown	95 (65.1%)

### Part of online questionnaire

**Figure 1.**
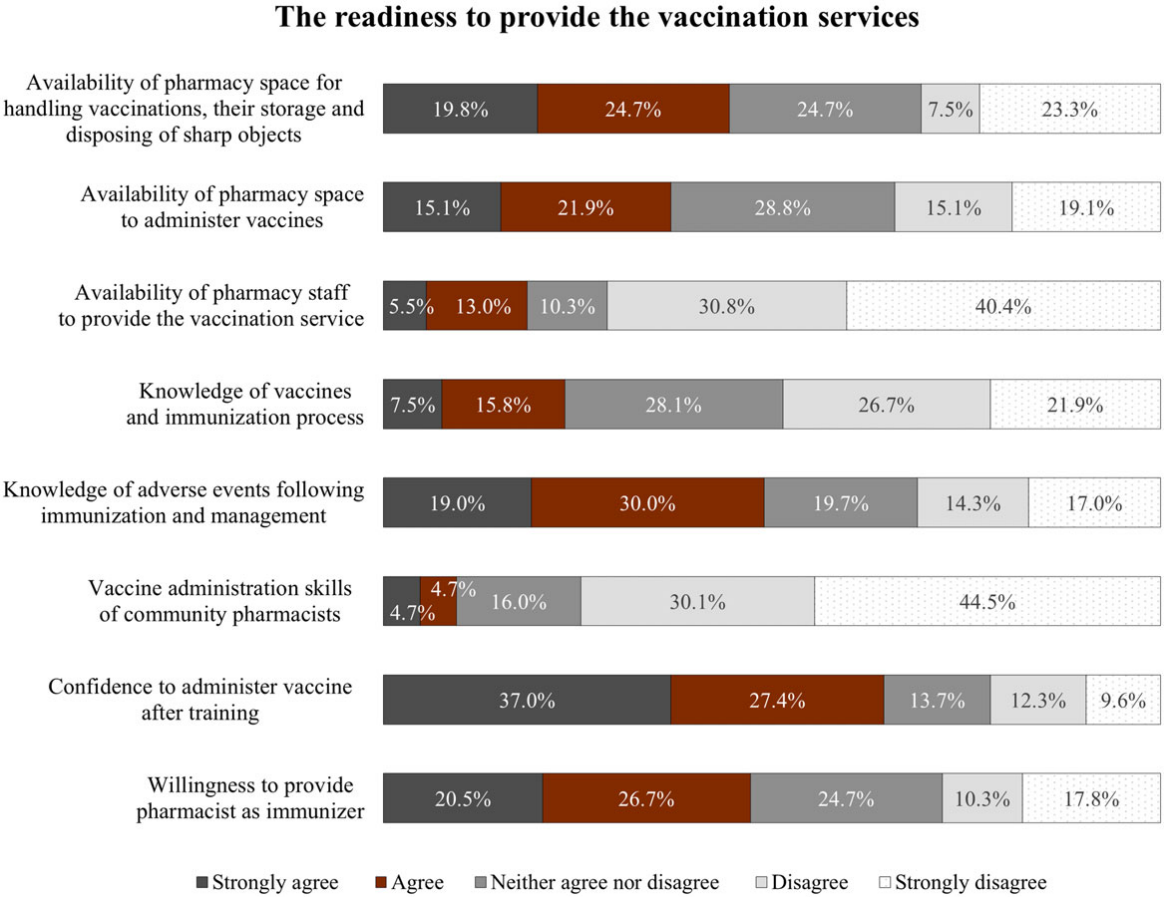
The readiness to provide the vaccination services.

The readiness of community pharmacies to provide the vaccination services is displayed in Figure 1. Almost 45% of respondents strongly agreed and agreed with the availability of pharmacy space for handling vaccinations, their storage, and disposing of sharp objects. Less than 10% of respondents had vaccine administration skills (4.7% strongly agreed and 4.7% agreed), and around 23% of respondents had knowledge of vaccines and the immunization process (7.5% strongly agreed and 15.8% agreed); however, 19.0% of respondents strongly agreed and 30.0% agreed with knowledge of AEFI and management. For the willingness to provide the vaccination service, some respondents confidently administered vaccine after receiving adequate training (37.0% strongly agreed and 27.4% agreed) and were willing to be pharmacists as immunizers (20.5% strongly agreed and 26.7% agreed).

More than 50% of respondents strongly agreed or agreed that community pharmacists can play the role of immunizers (Table 2). Almost all pharmacists agreed that vaccination services in community pharmacies are easily accessible to patients (39.7% of respondents strongly agreed and 25.3% agreed) and that vaccinations administered in community pharmacies will improve the overall rate of vaccination (38.4% of respondents strongly agreed and 30.8% agreed). More than 75% of respondents agreed that certified training in vaccine administration should be required for pharmacists before providing the service. However, most of the respondents were concerned about conflicts with other professionals qualified to administer vaccinations and the cost of setting up and service management. More than 50% of respondents were required to support remuneration and promote vaccination services in community pharmacies.

**Table 2.** The community pharmacists’ opinion of providing the vaccination services (*n* = 146)

The opinion^*^	Strongly agree	Agree	Neither agree nor disagree	Disagree	Strongly disagree
Community pharmacists can play the role of immunizers	33 (22.6%)	41 (28.1%)	32 (21.9%)	18 (12.3%)	22 (15.1%)
The community pharmacy is suitable to provide the vaccination service	17 (11.6%)	21 (14.4%)	35 (24.0%)	34 (23.3%)	39 (26.7%)
The vaccination service in community pharmacies is easily accessible to patients	58 (39.7%)	37 (25.3%)	15 (10.3%)	15 (10.3%)	21 (14.4%)
The vaccination service in community pharmacies reduce overcrowding in hospitals	57 (39.0%)	30 (20.5%)	29 (19.9%)	15 (10.3%)	15 (10.3%)
The vaccinations administered in community pharmacies will improve the overall rate of vaccination	56 (38.4%)	45 (30.8%)	18 (12.3%)	8 (5.5%)	19 (13.0%)
The vaccination services in community pharmacies promote the role of pharmacists for patients	49 (33.6%)	44 (30.1%)	26 (17.8%)	12 (8.2%)	15 (10.3%)
The vaccination service will add more work for community pharmacists	47 (32.2%)	52 (35.6%)	29 (19.9%)	12 (8.2%)	6 (4.1%)
The vaccination services in community pharmacies increase the cost of setup and management	59 (40.4%)	37 (25.3%)	33 (22.6%)	7 (4.8%)	10 (6.8%)
The government should support adequate remuneration	63 (43.1%)	43 (29.5%)	15 (10.3%)	10 (6.8%)	15 (10.3%)
The government should promote vaccination services in community pharmacies for patients	43 (29.6%)	37 (25.3%)	30 (20.5%)	18 (12.3%)	18 (12.3%)
Conflicts with other professionals qualified to administer vaccinations are likely to occur	34 (23.3%)	39 (26.7%)	40 (27.4%)	18 (12.3%)	15 (10.3%)
Certified training in vaccine administration should be required for pharmacists before providing the service	98 (67.1%)	14 (9.6%)	8 (5.5%)	15 (10.3%)	11 (7.5%)

* Data showed as number (%).

**Figure 2.**
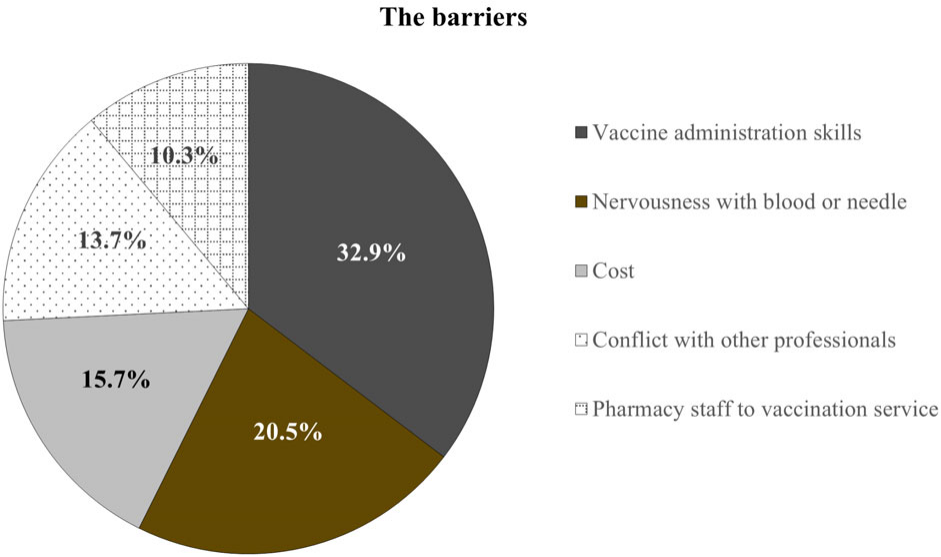
The barriers to provide the vaccination services.

The barriers to provide the vaccination services is shown in Figure 2. The barriers most frequently identified were vaccine administration skills (32.9%), nervousness with blood or needle (20.5%), and cost (15.7%).

### Part of a semi-structured interview

A total of 12 community pharmacists agreed to be interviewed, and all of the interview respondents were pharmacy owners. Three themes of pharmacists’ perception of vaccination services in community pharmacies were benefits, concerns, and proposing.

#### Benefits

Respondents stated that the patients trust and recognize the greater role of the community pharmacist, and the community pharmacy may become better known because of this service.

*“Vaccination service in community pharmacy can expand the pharmacist role. Almost all patients think that the community pharmacist only prescribes medication, followed by the patient’s symptoms.” (Pharmacist No 2)*

*“This service shows the patient that the role of the new generation of community pharmacists is different from that of the old community pharmacists.” (Pharmacist No 5)*

*“This service is an opportunity to get new customers for the pharmacy.” (Pharmacist No 8)*

Respondents expressed that patients visit the community pharmacy for vaccinations, which is convenient, reduces the waiting time and reduces the burden on doctors and nurses. Patients increase access to vaccination because of this service.

*“Patients find it easy and comfortable to assess the vaccine in the community pharmacy.” (Pharmacist No 1)*

*“This service improves the patient’s comfort, which is all service in the pharmacy.” (Pharmacist No 3)*

*“…easy and rapid to assess the vaccines in the community pharmacy, especially in the pandemic situation. The community pharmacy distributes all areas, and the patient can assess their first.” (Pharmacist No 5)*

*“Vaccines administered by community pharmacists might reduce the workload of doctors and nurses.” (Pharmacist No 6)*

#### Concerns

Respondents stated that vaccination administration by pharmacist may duplicate the roles of other professions. The details of the vaccination service policy are not adequate to encourage the community pharmacist to enroll in this service.

*“Vaccine administration is the role of doctors and nurses who have training and practice in the faculty classes.” (Pharmacist No 8)*

*“Pharmacists have knowledge of vaccination but not the skill for administration. The duration of the training program by the Pharmacy Council of Thailand is too short, not enough to build the confidence of the pharmacist and patient.” (Pharmacist No 2)*

*“The policy of vaccination service in the community pharmacy is not definitely a program since vaccine supply, follow-up with the patient, law, and reimbursement.” (Pharmacist No 11)*

#### Proposing

Respondents expressed that they need to know more details about the vaccination service policy, and vaccine administration skills should be taught in faculty classes

*“The Pharmacy Council of Thailand should design the criteria of the vaccine that is available in community pharmacies, such as the well-known vaccine or a few adverse effects.” (Pharmacist No 5)*

*“Vaccine administration skills can be practiced; they are not the barrier. If the community pharmacist is not confident in this skill, they can contact the nurse to do it for the first time.” (Pharmacist No 4)*

*“…should enroll in the training in the classes.” (Pharmacist No 9)*

*“For the beginning, the service starts the common vaccine or the necessary vaccine in the pandemic situation in adults.” (Pharmacist No 1)*

## Discussion

This study examined pharmacists’ readiness, barriers, and opinions for vaccination services in community pharmacies. According to the policy for pharmacist immunizers involved with the National List of Essential Vaccines, our findings showed that approximately 45% of the surveyed community pharmacists were willing to be immunizers, there was readiness of pharmacy space, knowledge of AEFI, and management for vaccination service. Their opinion agreed that community pharmacists can play the role of immunizers and improve the overall rate of vaccination.

The willingness of community pharmacists to immunize in our study was lower than in another study. Kristina *et al.* showed that 80.0% of community pharmacists were willing to administer the COVID-19 vaccine in Indonesia (Kristina *et al.*, 2022), 66.5% were willing in Lebanon (Youssef *et al.*, 2021), and more than 85% had a high level of willingness in Jordan and New Zealand (Gauld *et al.*, 2020; Jarab *et al.*, 2022). In our study, the reasons for the lower rate of willingness were: 1. there is information on the cold chain delivery system and vaccines but lack of vaccine administration skills in the faculty classes, which was the most important factor in the willingness to study of Turcu *et al.* and Ang et al (Turcu-Stiolica *et al.*, 2021; Ang *et al.*, 2022), 2. this is the new role of Thai pharmacists, so they may not be sure about the conflict with other professionals and the trend of policy, and 3. we studied in the urban region so that the patient could easily assess the vaccines in the hospital or private clinic. Ang *et al.* found the community pharmacist’s willingness to provide vaccination services was higher in the rural region (Ang *et al.*, 2022).

Our study revealed that pharmacists have a positive attitude toward immunization. The community pharmacists believed that vaccination services in community pharmacies are easily accessible to patients and improve the overall rate of vaccination. Another study showed that vaccination coverage rates were higher when pharmacists provided vaccinations because of enhanced accessibility and convenience (Isenor *et al.*, 2018; Youssef *et al.*, 2021). However, some of the respondents in this study were concerned about workload, like in another study (Gauld *et al.*, 2020; Merks *et al.*, 2021; Jarab *et al.*, 2022).

The main barrier to vaccination service provision in our study, similar to that of an online survey and interviewing, was knowledge and skills of vaccine administration, as was the case with the study in Indonesia and Malaysia (Ang *et al.*, 2022; Kristina *et al.*, 2022). Community pharmacists in Turkey had a low level of knowledge about vaccination during pregnancy (Ozdemir *et al.*, 2022) but lack of vaccine knowledge was not shown as a barrier in the several study (Kamal *et al.*, 2003; Valiquette and Bédard, 2015; Merks *et al.*, 2021). While other studies showed that lack of authorization and reimbursement were important barriers (Isenor *et al.*, 2018; Jarab *et al.*, 2022; Lindner *et al.*, 2022), few respondents in this study concerned about these barriers. The non – trained pharmacists have shown more barriers to providing the service than the training pharmacists (Merks *et al.*, 2021; Ang *et al.*, 2022). Due to the lack of training in faculty classes, as mentioned above, and the community pharmacists’ role in Thailand’s only vaccine education and engagement program. They need for and are aware of the importance of vaccine administration training before providing services, since the Pharmacy Council of Thailand has just launched the training program at the time of the survey. The training includes two days of lectures on the National List of Essential Vaccines for pediatric, adult, and special population; AEFI management; vaccine storage and cold chain systems; and practical skills in vaccine administration. Concern about cost was the main barrier in New Zealand (Gauld *et al.*, 2020); however, only some of the respondents in this study concerned about this subject, possibly because the vaccines in the National List of Essential Vaccines were supported by the government. The maintenance of the cold chain at the point of vaccine arrival, storage, handling, and administration are important aspects of vaccination service. Lack of space for vaccination services was the barrier in some studies (Isenor *et al.*, 2018; Turcu-Stiolica *et al.*, 2021; Jarab *et al.*, 2022; Alotaibi *et al.*, 2023), but not included in this study, as almost 45% of the respondents self-evaluated the availability of pharmacy space, similar to study in Saudi Arabia that most of the pharmacists showed positive attitude towards the vaccination strategy by community pharmacists (Alshahrani *et al.*, 2022).

### Strengths and limitations

The study shows the readiness and barriers of community pharmacists as immunizers. These data should be used by the government to develop policies and support community pharmacies for vaccination services. The limitation of this study was the small sample size only in Hatyai, Songkhla province. The future study should be conducted for a national survey. Another limitation might be selection bias from the online questionnaire, which allows community pharmacists to have access to online resources. In addition to this study, we should survey patients’ perspectives on vaccination services in community pharmacies.

### Implications for practice

The vaccine supply and logistics system are assigned to the Government Pharmaceutical Organization (GPO) then distributed to the primary care unit, or health center where immunizations take place (Kategeaw *et al.*, 2022). Community pharmacies should be a part of the process. The vaccine should be stored in community pharmacies only when an appointment is available. Vaccine administration skills should be trained in faculty classes and routinely recertified. To avoid conflict of interest, the vaccines on the National List of Essential Vaccines that are supported by the government should be started in community pharmacies.

## Conclusion

The community pharmacists had a positive perception of the role of pharmacists as immunizers. The vaccinations administered in community pharmacies are easily accessible to patients and improve the overall rate of vaccination. The readiness of community pharmacies to provide vaccination services was pharmacy space and knowledge of AEFI. Most of the concerns were vaccine administration skills, the conflict with other professionals, and the cost of setup and management. The pharmacists required training in vaccine administration skills before providing the service.

## Data Availability

The data that support the findings of this study are available on request from the corresponding author. The data are not publicly available due to privacy or ethical restrictions.
